# Serum Metabolome Signatures Characterizing Co-Infection of *Plasmodium falciparum* and HBV in Pregnant Women

**DOI:** 10.3390/diseases11030094

**Published:** 2023-07-05

**Authors:** Gloria Asantewaa, Nsoh Godwin Anabire, Michael Bauer, Sebastian Weis, Sophie Neugebauer, Osbourne Quaye, Gideon Kofi Helegbe

**Affiliations:** 1 West African Centre for Cell Biology of Infectious Pathogens, Department of Biochemistry, Cell & Molecular Biology, University of Ghana, Accra P.O. Box LG54, Ghana; gloria_asantewaa@urmc.rochester.edu (G.A.); terrodeang@gmail.com (N.G.A.); oquaye@ug.edu.gh (O.Q.); 2 Department of Biochemistry & Molecular Biology, School of Medicine, University for Development Studies, Tamale P.O. Box TL1350, Ghana; 3 Department of Anesthesiology and Intensive Care Medicine, Jena University Hospital, Friedrich-Schiller University, 07747 Jena, Germany; michael.bauer@med.uni-jena.de (M.B.); sebastian.weis@med.uni-jena.de (S.W.); 4 Center for Sepsis Control and Care (CSCC), Jena University Hospital, Friedrich-Schiller University, 07747 Jena, Germany; 5 Institute for Infectious Disease and Infection Control, Leibniz Institute for Infection Biology and Natural Product Research, Hans-Knöll Institute (HKI), 07745 Jena, Germany; 6 Leibniz Institute for Natural Product Research and Infection Biology, Hans-Knöll Institute (HKI), 07745 Jena, Germany; 7 Institute of Clinical Chemistry and Laboratory Diagnostics, Jena University Hospital, 07747 Jena, Germany; sophie.neugebauer@med.uni-jena.de

**Keywords:** malaria, hepatitis B, pregnancy, serum metabolites, co-infection

## Abstract

*Plasmodium falciparum* (*P. falciparum*) and hepatitis B virus (HBV) co-infection is on the rise among pregnant women in northern Ghana. Mono-infection with either of these two pathogens results in unique metabolic alterations. Thus, we aimed to explicate the effects of this co-infection on the metabolome signatures of pregnant women, which would indicate the impacted metabolic pathways and provide useful prognostic or diagnostic markers. Using an MS/MS-based targeted metabolomic approach, we determined the serum metabolome in pregnant women with *P. falciparum* mono-infection, HBV mono-infection, *P. falciparum*, and HBV co-infection and in uninfected (control) women. We observed significantly decreased sphingolipid concentrations in subjects with *P. falciparum* mono-infection, whereas amino acids and phospholipids were decreased in subjects with HBV mono-infection. Co-infections were found to be characterized distinctively by reduced concentrations of phospholipids and hexoses (mostly glucose) as well as altered pathways that contribute to redox homeostasis. Overall, PC ae C40:1 was found to be a good discriminatory metabolite for the co-infection group. PC ae C40:1 can further be explored for use in the diagnosis and treatment of malaria and chronic hepatitis B co-morbidity as well as to distinguish co-infections from cases of mono-infections.

## 1. Introduction

Pregnancy-associated adapted immunity and metabolism puts women at higher risk when infected with pathogens including *P. falciparum* and viral hepatitis B (HBV). Among patients with febrile illness, the effects of disease conditions in cases of co-infection are more severe than that observed in mono-infection cases [1]. Increased susceptibility to infection, as well as increased disease severity, has been reported in pregnant women who are infected with various infectious pathogens including *Plasmodium* spp. and HBV [2,3].

Hepatitis B infection during pregnancy poses additional complications in pregnancy, including an increased risk of preterm delivery [4]. Although there has been a decline in *P. falciparum* infections in certain regions globally, some regions within sub-Saharan Africa still record high malaria transmission in pregnant women, which can have diverse pathological effects on the placenta and the unborn baby [5,6].

The incidence of *P. falciparum* and HBV co-infection is highly probable in regions with high endemicity of the individual infections. In Ghana, cases of malaria and chronic HBV co-infection are common among pregnant women. An increasing prevalence trend (from 0.7% to 1.7%) in cases has been reported in northern Ghana [7,8]. This coinfection has been reported in other regions as well. There is an apparent neglect of the impact of *P. falciparum* and HBV co-infection during pregnancy, considering that *P. falciparum* infection is curable. Thus, although the effects of these mono-infections during pregnancy are well documented, there is a dearth of knowledge on the maternal and fetal effects of this co-infection during pregnancy. We have previously reported increased concentrations of liver damage biomarkers in the sera of pregnant women suffering from malaria and chronic hepatitis B co-infection, which thereby resulted in a significantly increased release of pro-inflammatory mediators in these women [9]. Liver dysfunction during pregnancy has been found to result in poor maternal and fetal outcomes [10]. Considering that pregnant women living in regions endemic to both malaria and HBV are at risk of acquiring a co-infection and the potential effects of this co-infection on fetal development, the timely diagnosis and treatment of *P. falciparum* and HBV co-infection are of paramount essence. 

The liver is responsible for most of the secreted metabolites circulated through the blood [11]. Thus, measuring metabolites in the serum gives a good indication of the metabolic state and integrity of the liver. Utilizing this approach herein, we aimed to evaluate the impact of *P. falciparum* and chronic HBV co-infection on serum metabolome profiles within the cohort of pregnant women. This is to elucidate the metabolic shifts and alterations that occur because of the altered immune response and liver damage in pregnant women with co-infection. 

## 2. Materials and Methods

### 2.1. Study Area and Participants

Sera samples used in this study were collected from pregnant women in a previous study [8]. Study participants were recruited from six sampling sites in different parts of northern Ghana, where we have previously reported an increasing trend in the prevalence of co-infection of *P. falciparum* and chronic HBV among pregnant women [7,8]. Women were recruited from Tamale Teaching Hospital, Tamale West Hospital, Tamale Central Hospital, and Bilpella Health Centre in the Tamale metropolis of northern Ghana. In the Central Gonja district of northern Ghana, the sampling sites included Sankpala Health Centre and Kosawgu Health Centre [9]. Women were recruited at random on their first antenatal visit between October 2016 and February 2017. Pregnant women who had been diagnosed with preeclampsia/eclampsia, degenerative diseases, sickle cell trait, or viral infections except for hepatitis B were excluded from the study. Similarly, participants with documented alcoholism as well as those who were on either immunosuppressants or hepatotoxic drugs were excluded from the study.

The obstetric data of the study participants are reported in Table 1.

### 2.2. Serum Samples

The description and categorization of sera samples into infection groups were performed as previously reported [9]. Sera samples from pregnant women that tested positive via PCR for *P. falciparum*, HBV, or their co-infection were used for metabolome analysis in this study. Additionally, sera were obtained from women who were uninfected with the two pathogens and used as controls. Sera were stored in cryo-tubes at −80 °C until metabolome analysis.

### 2.3. Ethical Consideration

Ethical approval was sought from the Tamale Teaching Hospital Ethical Committee (approval ID: TTHERC/21/04/16/02) and the Navrongo Health Research Centre Institutional Review Board (approval ID: NHRCIRB4O2). Written informed consent was obtained from each study participant. 

### 2.4. Metabolite Profile Analysis

The AbsoluteIDQ^®^p180 kit (Biocrates Life Science AG, Innsbruck, Austria) was used for targeted metabolomic analysis. The concentration of 188 metabolites from 6 biochemical classes (21 amino acids, 14 lysophosphatidylcholines, 21 biogenic amines, 1 hexose, 40 acylcarnitines, 76 phosphatidylcholines, and 15 sphingolipids) were measured in serum according to the manufacturer’s protocol on an API4000 LC-MS/MS-System (AB Sciex, Framingham, MA, USA) equipped with an Agilent 1200 Series HPLC System (Waters, Milford, MA, USA), a CTC PAL autosampler (CTC Analytics AG, Zwingen, Switzerland), and the Analyst 1.6.2 software (AB Sciex). In brief, 10 µL of the internal standards were added onto the center of the spots in the kit plate and dried using a nitrogen evaporator for 15 min at room temperature. In a Laminar Air Flow Box, 10 µL of calibration standards, quality control, zero sample or study sample were also added and incubated until the samples dried. Subsequently, 50 µL of a 5% solution of phenylisothiocyanate (Merck, Darmstadt, Germany) were pipetted onto the spots, incubated for 45 min at room temperature, and dried again using the evaporator for 1 h. The metabolites were extracted with 300 μL of a 5 mM ammonium acetate solution in methanol (Merck, Darmstadt, Germany; Roth, Karlsruhe, Germany) and incubated for 30 min in a shaker (450 rpm). The plate was centrifuged at 100× *g* for 2 min, and the upper plate was removed. Volumes of 150 μL of each sample were transferred to a second plate (LC-MS plate) and diluted with 150 µL HPLC-grade water. The remaining volume in the original plate (flow injection analysis (FIA) plate) was diluted 1:4 with FIA solvent (Biocrates solvent diluted in methanol). The LC-MS plate was measured first via multiple reaction monitoring, and the FIA plate was stored at 4 °C. A volume of 10 µL of the sample was injected onto a Zorbax Eclipse XDB C18 3 × 100 mm column (Agilent) coupled to a C18 4 × 3 mm security guard precolumn (Phenomenex) and eluted with solvent A (HPLC water + 0.2% formic acid) and solvent B (acetonitrile + 0.2% formic acid). For analysis of the FIA plate, two subsequent 20 μL injections of FIA solvent were again injected directly into the MS at a flow rate 30 μL/min. Concentrations for metabolites were determined using the MetIDQ™ software package, which is an integral part of the AbsoluteIDQ^®^p180 kit, by comparing measured analyte/internal standard ratios or measured analytes in a defined extracted ion count section to those of the calibration curve, specific labeled internal standards, or nonlabelled, nonphysiological standards (semiquantitative) provided by the kit plate. These data were then normalized sample-wise with quality control and exported for subsequent statistical analysis.

### 2.5. Data Processing and Statistical Analysis

Metaboanalyst 5.0 [12] was used in the data analysis. The full list of analyzed metabolites and their full names are indicated in the Supplementary Data (Supplementary Table S1). Missing values were imputed using the k-Nearest-Neighbors (KNN) method. The data were then normalized via autoscaling. Principal Component Analysis (PCA) and Partial Least Square-Discriminant Analysis (PLS-DA) were carried out on the data set to uncover both outliers and the different groups’ distributions. Next, a *t* test was used to initially assess significant metabolites. The Benjamini–Hochberg false discovery rate (FDR) correction method was employed to correct the errors of multiple hypothesis testing with an FDR (false discovery rate) cutoff of 0.05. These were further validated using the Variable Importance for Projection (VIP) feature of PLS-DA. A VIP score > 1.5 annotation was also used to qualify the metabolites. Biomarker analysis was then further carried out on significant metabolites identified from either PLS-DA or *t* tests. The biomarker analysis was performed using receiver operating characteristic (ROC) analysis, with Area Under the Curve (AUC) values > 0.7 being considered significant. Additionally, the metabolic data was also analyzed using Metabolite Set Enrichment Analysis (MSEA) to extract pathways that were particularly enriched due to the metabolic signatures in the groups. Figures were generated using Metaboanalyst 5.0 and GraphPad Prism 9.

## 3. Results

### 3.1. Sample Characterization

**Figure 1:**
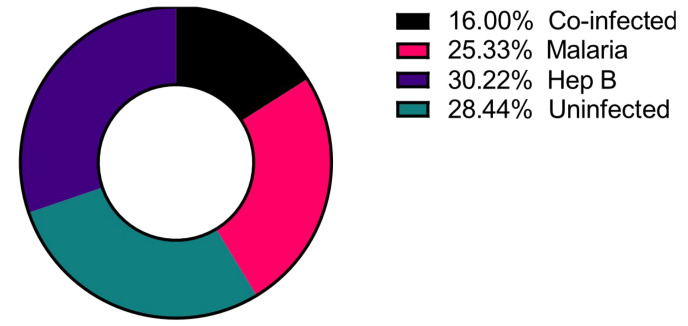
Distribution of the disease categories used in the study. Number of study participants = 225.

A total of 225 samples from the northern region of Ghana were used, and of these, 36 (16.0%) had co-infections of *P. falciparum* and HBV (Co-infected), 57 (25.0%) were *P. falciparum* mono-infected (Malaria only), and 68 (30.0%) were HBV mono-infected (Hep B only) as determined via PCR (Figure 1) [9]. These groups were compared to 64 (29.0%) control samples that tested negative for infection via PCR for both pathogens (Uninfected). 

### 3.2. P. falciparum Infection Alters Sphingolipid Serum Concentrations of Pregnant Women

**Figure 2:**
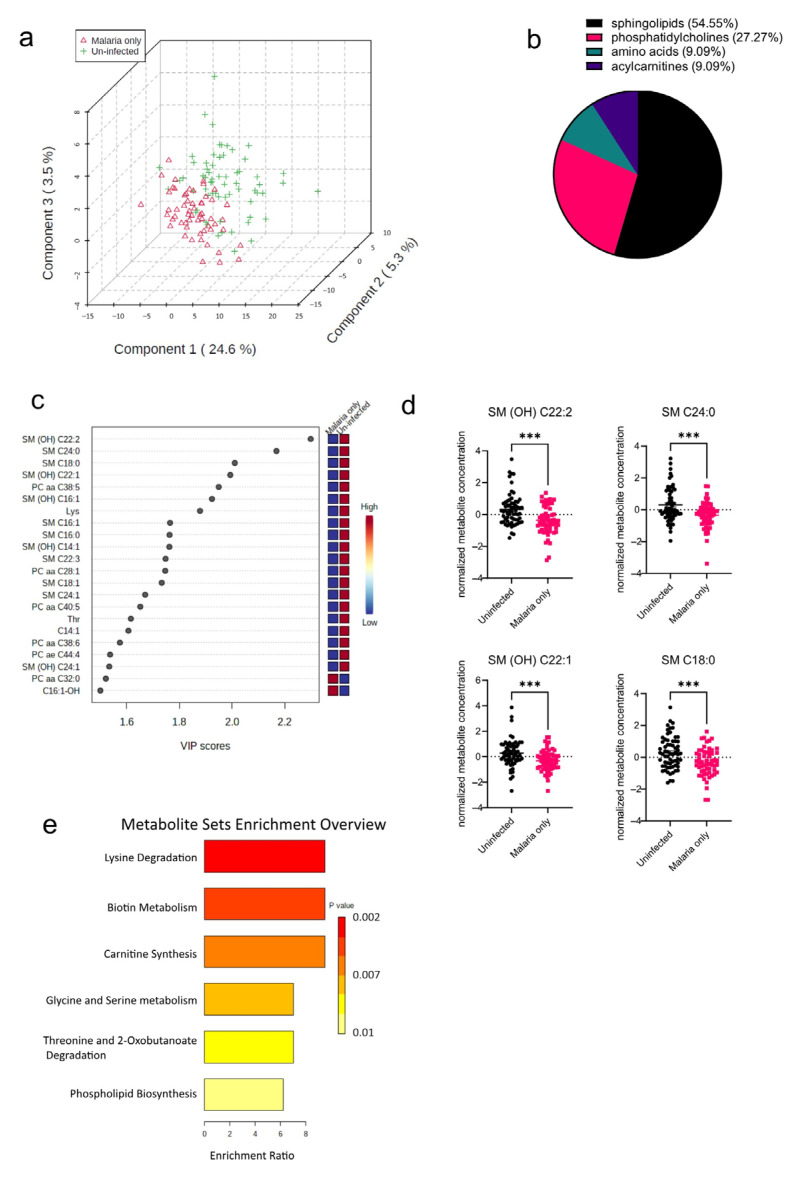
Significant metabolites detected in the malaria group. (**a**) Three-dimensional score plot between the first three components showing the clustering of the Malaria vs. Uninfected samples. The described variances are indicated on the axes and in the brackets. (**b**) Pie chart indicating the percentage of significantly altered metabolite classes. (**c**) VIP score plot showing the significantly altered metabolites in the Malaria vs. Uninfected samples. The attached pseudo-heat maps on the right signify the various concentrations of each corresponding metabolite in its disease group. (**d**) Scatter plots of the four most altered serum metabolites in the Malaria group compared to the Uninfected group that show the metabolite concentrations in each sample. Data on the graph represent means ± SEM. *** *p* value < 0.001. (**e**) Summary plot for Quantitative Enrichment Analysis (QEA) showing the enrichment ratios of metabolic pathways in the Malaria serum samples compared to the Uninfected samples.

The pathophysiology of malaria diverges in pregnant cases, which implies that infected pregnant women are at an increased risk of infection severity and its complications [13]. As a result of the increased risk of malaria morbidity, the serum metabolome signature in *P. falciparum*-infected pregnant women was determined and compared to that of uninfected pregnant women. Principal component analysis (PCA) was performed to visualize how well the samples separated based on the disease groups (Malaria vs. Uninfected). The PCA plot shows that in addition to the variation based on the disease groups, there were also some observed levels of inter-individual variations within the groups as well (Figure 2a). *T* tests and PLS-DAs accounting for false discovery rates were also performed to identify metabolites significantly altered in the malaria mono-infected group compared to the uninfected group. From these analyses, a total of 22 out of the 188 metabolites analyzed were found to be significantly altered (Figure 2b), with 12 metabolites (54.55%) out of the 22 being sphingolipids (Figure 2b). Only two of these metabolites, PC aa C32:0 (phospholipid) and C16:1-OH (acylcarnitine), were increased in the malaria group; the rest including the oxidized metabolites, indicated with “(OH)”, were all found to be markedly decreased in the malaria group (Figure 2c). The most significantly altered metabolites in the malaria group were the sphingolipids SM (OH) C22:2, SM C24:0, SM C18:0, and SM (OH) C22:1 (Figure 2d). To identify the various pathways impacted, the significantly altered metabolites were further analyzed using a hypergeometric test, an over-representation analysis method. Among the malaria group, it was found that the most enriched metabolic pathways were related to lipid and amino acid metabolism pathways, such as those of phospholipid metabolism and serine metabolism (Figure 2e). Because serine is one of the primary components required for the synthesis of sphingolipids [14], its increased metabolism augments or complements the significantly altered sphingolipid species identified in the univariate analysis.

### 3.3. Pregnant Women Infected with HBV Have Altered Amino Acid and Phospholipid Concentrations

**Figure 3:**
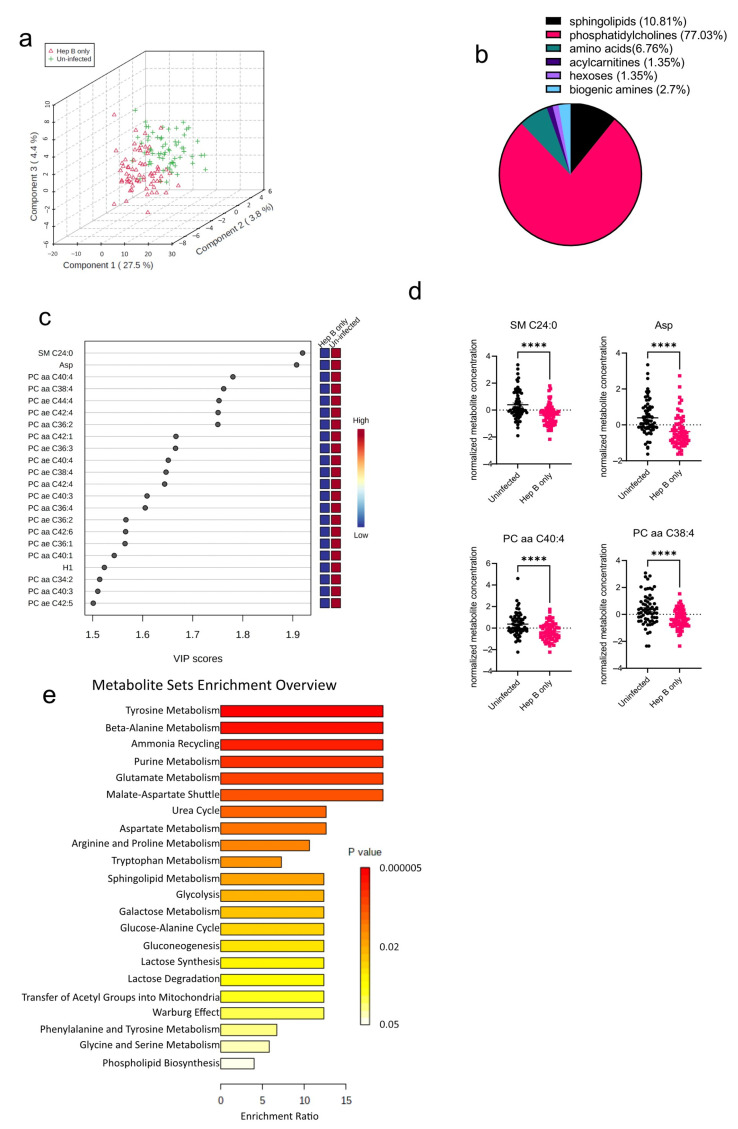
Significant metabolites detected in the hepatitis B (Hep B) group. (**a**) Three-dimensional score plot between the first three components showing the clustering of Hep B (hepatitis B) and Uninfected samples. The described variances are indicated on the axes and in the brackets. (**b**) Pie chart indicating the percentage of significantly altered metabolite classes in the Hep B vs. Uninfected sample groups. The total number of altered metabolites found = 74. (**c**) VIP score plot showing the top 22 significantly altered metabolites in the Hep B group vs. the Uninfected group. The attached pseudo-heat maps on the right signify the various concentrations of each corresponding metabolite in its disease group. (**d**) Scatter plots of the four most altered metabolites in the Hep B vs. Uninfected groups, showing their normalized concentrations in each sample group. Data on the graph represent means ± SEM. **** *p* value < 0.0001. (**e**) Summary plot for Quantitative Enrichment Analysis (QEA) in the Hep B vs. Uninfected groups that shows enriched metabolic pathways.

The algorithms used in identifying the significantly altered metabolites in the malaria group were also employed in the hepatitis B group as well. An initial PCA analysis revealed that like the malaria group, there was some overlap between the hepatitis B group (Hep B only) and the uninfected group (Uninfected) (Figure 3a). Upon analysis of the altered metabolites in the hepatitis B group, 74 metabolites were found to be significantly altered (Figure 3b). The 74 significantly altered metabolites in the hepatitis B group, compared to only 22 altered metabolites found in the malaria group, could be due to the fact that during infection, HBV majorly occupies the liver, the major metabolic organ of the body, and causes more liver damage, whereas *P. falciparum* only inhabits hepatocytes at certain stages of its life cycle [15,16]. Of the 74 significantly altered metabolites, phosphatidylcholines were the most significantly altered metabolite group (Figure 3b). All of the 22 most-altered metabolites were found to be lowered in the hepatitis B group (Figure 3c). The most significantly reduced metabolites in the hepatitis B group were SM C24:0, a sphingolipid, and aspartate, an amino acid (Figure 3d). Aspartate concentrations in the body are predominantly regulated by aspartate aminotransferase, a liver damage biomarker previously reported by our group to be severely altered in the serum of hepatitis B-infected pregnant women [9]. Based on the aforementioned identified metabolites, further enrichment analysis was also done to explore the metabolic pathways most impacted in the hepatitis B group (Figure 3e). As expected, metabolic pathways involving phospholipid biosynthesis were found to be altered. In addition, most of the significantly enriched pathways were those involving amino acid metabolism such as those for glycine, serine, and lysine.

### 3.4. Co-Infection of P. falciparum and HBV Induces Reduced Metabolite Serum Concentrations in Pregnant Women

**Figure 4:**
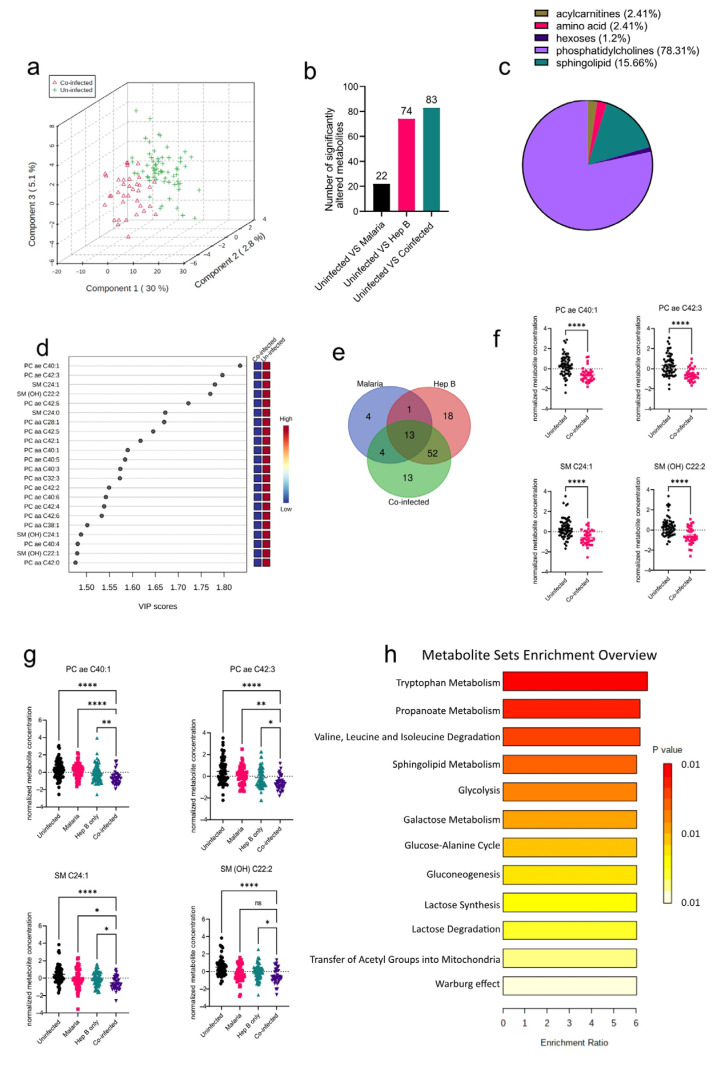
Significant metabolites detected in the co-infected group. (**a**) Three-dimensional score plot between the first three components that shows the clustering of the co-infected vs. Uninfected sample groups. The described variances are indicated in the axes and in the brackets. (**b**) Bar chart indicating the number of significantly altered metabolites in all of the disease groups. Each bar is annotated with the number of metabolites for that particular disease group. (**c**) Pie chart indicating the percentage of significantly altered metabolite classes in the co-infected vs. Uninfected sample groups. (**d**) VIP score plot showing the top 22 significantly altered metabolites in the co-infected vs. Uninfected groups. The attached pseudo-heat maps on the right signify the various concentrations of each corresponding metabolite in its disease group. (**e**) Venn diagram showing significantly altered metabolites found to be common among the various disease groups. Each set is annotated with its number of metabolites. (**f**) Scatter plots of the four most altered metabolites in the co-infected vs. Uninfected groups that show the normalized concentrations in each sample group. Data on the graph represent means ± SEM. **** *p* value < 0.0001. (**g**) Scatter plots of the four most-altered metabolites in the co-infected group compared to the malaria and hepatitis B groups that show their normalized concentrations in each sample group. Data on the graph represent means ± SEM. ns = not significant, * *p* value < 0.05, ** *p* value < 0.01, **** *p* value < 0.0001. (**h**) Summary plot for Quantitative Enrichment Analysis (QEA) of the metabolic pathways enriched in the co-infected vs. Uninfected groups.

After establishing the metabolites altered in the mono-infection groups and the corresponding pathways affected, the sera of pregnant women who had *P. falciparum* and HBV co-infection (co-infected) were analyzed to determine unique metabolic profiles. Compared to the PCA analysis from the mono-infected groups, the co-infected vs. Uninfected samples were better separated into their groups (Figure 4a), indicating a unique metabolic profile in the co-infected group induced by the co-infection of the parasites. Interestingly, the co-infected group yielded more significantly altered metabolite classes from the Uninfected group than any of the mono-infected groups (Figure 4b). Comparison of the co-infected group to the Uninfected group yielded six significantly altered metabolite classes that predominantly comprised phosphatidylcholines and sphingolipids (Figure 4c). These were mostly found to be reduced in the co-infected groups (Figure 4d). Further evaluation of the altered metabolites in the various disease groups revealed that there were some commonly altered metabolites among the three disease groups. Out of the 188 metabolites analyzed, only 13 were found to be commonly altered in all three disease groups, which proves the heterogeneity of the three disease groups (Figure 4e). However, most of the altered metabolites in the co-infected group were found to be altered in the Hep B group as well (Figure 4e; Supplementary Table S2). This is not surprising, as it might imply that the co-infected and hepatitis B groups share a dominant liver damage phenotype. Overall, the four topmost metabolites found to be altered in the co-infected group comprised metabolites whose concentrations were also altered in the hepatitis B group: PC ae C40:1, PC ae C42:3 SM C24:1, and SM(OH) C22:2 (Figure 4f). Both SM C24:1 and SM(OH) C22:2 were altered in the malaria group as well. As a result of these overlapping metabolites across the disease groups (Figure 4e,f), the alterations in the concentrations of these metabolites were analyzed across all disease groups. Even though all four metabolites were found to be reduced in all disease groups compared to the uninfected, they were markedly more reduced in the co-infected group compared to the mono-infected groups, thereby further elaborating that the co-infected group had a more aggravated phenotype compared to the mono-infected groups.

Tryptophan metabolism was found to be the most enriched pathway in the co-infected group. (Figure 4g). Other pathways associated with oxidative stress such as the Warburg effect, glycolysis, gluconeogenesis, and lactose metabolism were all found to be enriched. Glycolysis and gluconeogenesis have also previously been reported to be altered in the sera of *P. falciparum* patients as well [17]. Oxidative stress induces upregulation of the glycolytic pathway in a metabolic phenomenon known as the Warburg effect. Both *P. falciparum* and HBV infections have been associated with increased oxidative stress. Likewise, in *Plasmodium* infections, oxidative stress has been associated with alterations in nutrient availability, iron, and heme metabolism as part of the host’s defenses [18]. These impact the redox balance and induce oxidative stress, thereby accounting for the enriched redox metabolism pathways found in this study.

### 3.5. PC ae C40:1 Is a Good Biomarker for P. falciparum and HBV Co-Infection

**Figure 5:**
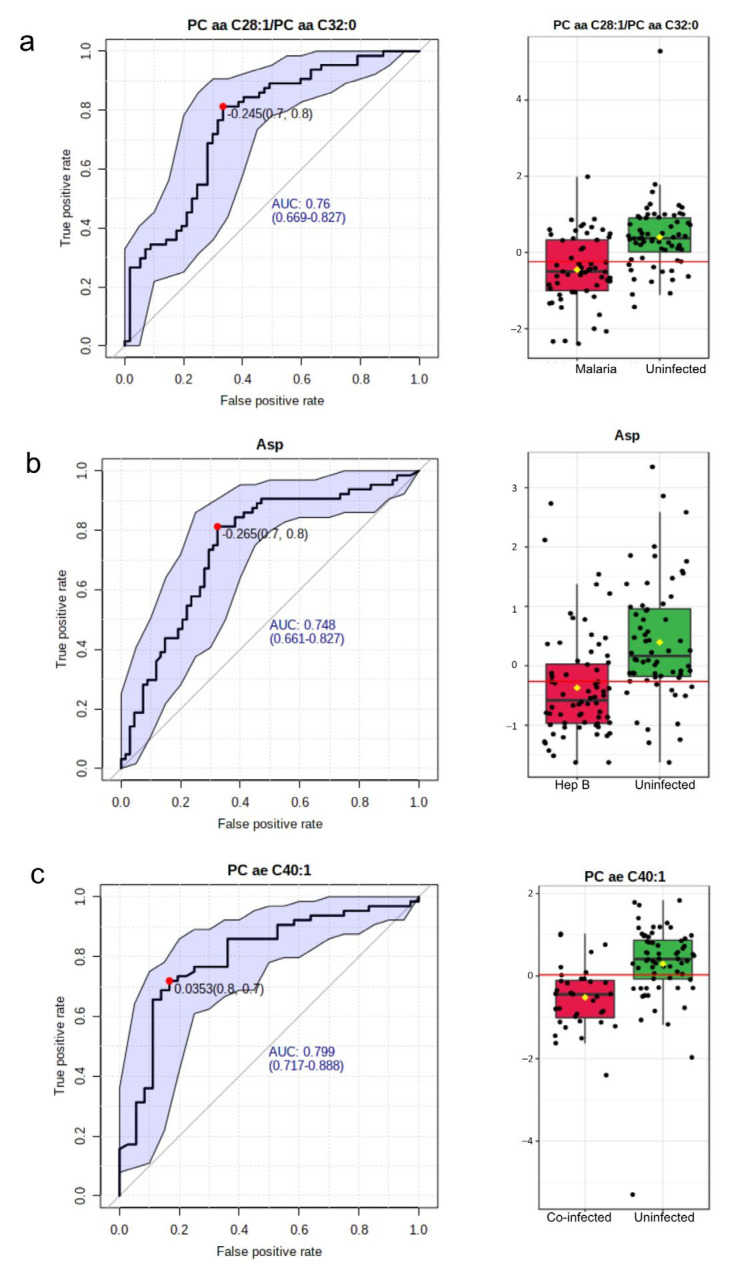
Biomarkers to characterize the disease groups. (**a**) Most significant potential biomarker in Malaria vs. Uninfected. Left: The ROC curve of PC aa C28:1/PC aa C32:0. Right: Boxplot of the concentrations of PC aa C28:1/PC aa C32:0 in the Malaria vs. Uninfected groups. (**b**) Most significant potential biomarker in the Hepatitis B group. Left: The ROC curve of Asp. Right: Boxplot of the concentrations of Asp in the hepatitis B group versus Uninfected. (**c**) Most significant potential biomarker in the co-infected versus Uninfected group. Left: The ROC curve of PC ae C40:1. Right: Boxplot of the concentrations of PC ae C40:1 in the co-infected group versus the Uninfected group. In all ROC curves, specificity is depicted on the x-axis, and sensitivity is depicted on the y-axis. The blue text in the graph indicates the area under the curve (AUC). In the box plots, the horizontal line in red indicates the optimal cutoff. The yellow diamond indicates the mean concentration for each sample category. The black dots correspond to the concentrations of the chosen metabolite in every sample. The notch in each plot signifies the 65% confidence interval.

The area under the Receiver Operating Characteristic (ROC) curve was utilized in evaluating the significance of a metabolite as a good biomarker for the various disease groups. In the malaria group compared with the Uninfected group, 15 such potential biomarkers were identified (Supplementary Table S3). The most significant biomarker based on the Area Under the Curve (AUC) was the ratio of two phosphatidylcholines: PC aa C28:1/PC aa C32:0. This had an AUC value of 0.76 (Figure 5a). In the hepatitis B group, out of nine potential biomarkers identified (Supplementary Table S4), aspartate was found to be the most significant with an AUC of 0.75 (Figure 5b). A total of 56 potential biomarkers were identified in the co-infected group (Supplementary Table S5) with *p*-values < 0.01. The metabolites involved included phosphatidylcholines, acylcarnitines, and amino acids. The topmost potential biomarker was PC ae C40:1 (Figure 5c).

## 4. Discussion

The seroprevalence of *P. falciparum* and HBV co-infection have been on the rise in the northern parts of Ghana, and thus, this study aimed to address the effects of this co-infection on the metabolic profiles of pregnant women living in this region. Consequently, lipid metabolites were found to be the most significantly altered across all disease groups. 

In the malaria group, the most significantly altered metabolite group was the sphingolipids. Both oxidized and unoxidized sphingolipids were found to be markedly reduced. This has also been previously observed upon host invasion by *P. falciparum* [19]. This observed decrease has been attributed to possible sphingolipid degradation or internalization by the parasite [20]. Phosphatidylcholines (phospholipids), which also make up the majority of the *Plasmodium* spp. membrane, were also significantly perturbed in the malaria group. This is comparable to reported altered phospholipid concentrations during *Plasmodium* spp. infection [21]. Phosphatidylcholines have been found to promote the growth of the parasite in the liver stages [22]. In *Plasmodium* infection, both host defense mechanisms and parasite activities induce changes in metabolism that upset the host’s heme metabolism and redox homeostasis [23,24]. These imbalances have been associated with the oxidation of lipids and ferroptosis. Thus, the reduction in the availability of some lipid species observed could potentially be part of the host’s machinery to circumvent cell damage. 

The significantly altered concentrations of phosphatidylcholines observed in the hepatitis B group are similar to those previously reported in patients with the onset of chronic HBV infection [25]. The major component of HBV’s envelope and surface antigens is phosphatidylcholines. It comprises approximately 60% of the viral lipids [26]. As a result, during HBV infection and proliferation in the hepatocytes, it uses the phosphatidylcholines synthesized by the liver. This is a possible reason for the decrease in the levels of phosphatidylcholines because most of the blood phosphatidylcholines are sourced from the liver.

In the co-infected cases, phosphatidylcholines and sphingolipids were both greatly reduced. Reduced levels of phospholipids are associated with increased stress in the endoplasmic reticulum (ER) and apoptosis. ER stress positively correlates with oxidative stress. This phenomenon could be a result of *Plasmodium*-induced heme accumulation and redox imbalance [23]. Oxidative stress can also be fostered by the activities of pro-inflammatory cytokines, which have already been shown to be exacerbated in co-infected cases [9,27]. 

Overall, it is not surprising that the most significantly decreased metabolites in the co-infected group were the lipid molecules. Lipid abundance in conditions of oxidative stress and increased levels of ROS can lead to lipid peroxidation. This condition is detrimental to cells and can cause membrane and tissue damage as well as damage to nucleic acids. Thus, reduced levels of lipid molecules have been reported in conditions of oxidative stress as a mechanism to prevent tissue damage [28]. A similar phenomenon could, therefore, be at play in the co-infected group. The liver, which is the common niche for HBV and *P. falciparum*, is the major site of lipid synthesis; hence, the extra pressure of liver damage observed in this co-infection could impact hepatic lipid metabolism and result in the observed reduced concentrations of lipid species.

Although this study tackled most confounding factors, some were still outstanding. The FIA–MS/MS analysis used to detect the lipid levels in the serum samples is limited. This is because it cannot distinguish between fatty acids that are linked to a glycerol backbone. Thus, a signal detected using this platform could also possibly represent other isomeric/isobaric lipids. 

In conclusion, co-infection of the malaria parasite with HBV induces unique alterations in lipid metabolite concentrations in pregnant women compared to those infected with either *P. falciparum* alone or HBV. The data presented here indicate pregnant women with malaria and chronic hepatitis B co-infection have significantly lower levels of glycerophospholipid species as well as altered pathways that contribute to redox homeostasis compared to their mono-infected counterparts. The identified metabolic biomarker for *P. falciparum* and HBV co-infection was PC ae C40:1. Further validation studies among a more geographically diverse population of pregnant women could be used to further validate the accuracy of this biomarker.

## Figures and Tables

**Table 1:** Obstetric data distribution of study participants.

Sample Characteristics	Gestation Period			Gravidity	
	1st Trimester	2nd Trimester	3rd Trimester	Primigravida	Multigravida
Uninfected (*n* = 64)	23 (35.9%)	28 (43.8%)	13 (20.3%)	9 (14.1%)	55 (85.9%)
Malaria (*n* = 57)	19 (33.3%)	31 (54.4%)	7 (12.3%)	15 (26.3%)	42 (73.7%)
Hep B (*n* = 68)	31 (45.6%)	32 (47.1%)	5 (7.4%)	16 (23.5%)	52 (76.5%)
Co-infected (*n* = 36)	9 (25.0%)	26 (72.2%)	1 (2.8%)	9 (25.0%)	27 (75.0%)

## Data Availability

Data generated during this study are included in this published article and its supplementary information files. Metabolomic datasets are available from the corresponding author upon request.

## Supplementary Materials

The following supporting information can be downloaded at: https://www.mdpi.com/2079-9721/11/3/94/s1, Supplementary Table S1: List of Metabolites Analyzed in the Study; Supplementary Table S2: Venn diagram results showing the metabolite elements shared among the disease groups; Supplementary Table S3: Area Under the Curve (AUC) values for univariate biomarker analysis in malaria vs. Uninfected groups; Supplementary Table S4: Area Under the Curve (AUC) values for univariate biomarker analysis in the Hep B vs. Uninfected groups; Supplementary Table S5: Area Under the Curve (AUC) values for univariate biomarker analysis in the co-infected vs. Uninfected groups. Supplementary Table S6: Area Under the Curve (AUC) values for univariate biomarker analysis in the Co-infected vs. uninfected groups.
