# Supplementary material for: Serum Metabolome Signatures Characterizing Co-Infection of Plasmodium falciparum and HBV in Pregnant Women
Source: Diseases. 2023 Jul 5;11(3):94. doi: 10.3390/diseases11030094 (PMC10366841; doi:10.3390/diseases11030094)
Supplement: Supplementary file 1 [file diseases-11-00094-s001.zip › diseases-2429024-supplementary.pdf]

TITLE PAGE

Serum Metabolome Signatures Characterizing Co-Infection of *Plasmodium falciparum* and HBV in Pregnant Women

Gloria Asantewaa [1], Nsoh Godwin Anabire [1,2], Michael Bauer [3,4], Sebastian Weis [3,4,5,6], Sophie Neugebauer [7], Osbourne Quaye [1] and Gideon Kofi Helegbe [1,2,*]

[1] West African Centre for Cell Biology of Infectious Pathogens; Department of Biochemistry, Cell & Molecular Biology, University of Ghana, Accra P.O. Box LG54, Ghana; gloria_asantewaa@urmc.rochester.edu (G.A.); terrodeang@gmail.com (N.G.A.); oquaye@ug.edu.gh (O.Q.)

[2] Department of Biochemistry & Molecular Biology, School of Medicine, University for Development Studies, Tamale P.O. Box TL1350, Ghana

[3] Department of Anesthesiology and Intensive Care Medicine, Jena University Hospital, Friedrich-Schiller University, 07747 Jena, Germany; michael.bauer@med.uni-jena.de (M.B.); sebastian.weis@med.uni-jena.de (S.W.)

[4] Center for Sepsis Control and Care (CSCC), Jena University Hospital, Friedrich-Schiller University, 07747 Jena, Germany

[5] Institute for Infectious Disease and Infection Control, Leibniz Institute for Infection Biology and Natural Product Research, Hans-Knöll Institute HKI, 07745, Jena, Germany

[6] Leibniz Institute for Natural Product Research and Infection Biology, Hans-Knöll Institute (HKI), 07745 Jena, Germany

[7] Institute of Clinical Chemistry and Laboratory Diagnostics, Jena University Hospital, 07747 Jena, Germany; sophie.neugebauer@med.uni-jena.de

* Correspondence: ghelegbe@uds.edu.gh

SUPPLEMENTARY TABLES

Supplementary Table S1: List of Metabolites Analyzed in the Study

	Metabolite	Full Metabolite Name	Metabolite Class
1	**C0**	Carnitine	acylcarnitine
2	**C2**	Acetylcarnitine	acylcarnitine
3	**C3**	Propionylcarnitine	acylcarnitine
4	**C3-DC (C4-OH)**	Hydroxybutyrylcarnitine	acylcarnitine
5	**C3-OH**	Hydroxypropionylcarnitine	acylcarnitine
6	**C3:1**	Propenoylcarnitine	acylcarnitine
7	**C4**	Butyrylcarnitine	acylcarnitine
8	**C4:1**	Butenylcarnitine	acylcarnitine
9	**C5**	Valerylcarnitine	acylcarnitine
10	**C5-DC (C6-OH)**	Glutarylcarnitine (Hydroxyhexanoylcarnitine)	acylcarnitine
11	**C5-M-DC**	Methylglutarylcarnitine	acylcarnitine
12	**C5-OH (C3-DC-M)**	Hydroxyvalerylcarnitine (Methylmalonylcarnitine)	acylcarnitine

13	**C5:1**	Tiglylcarnitine	acylcarnitine
14	**C5:1-DC**	Glutaconylcarnitine	acylcarnitine
15	**C6 (C4:1-DC)**	Hexanoylcarnitine (Fumarylcarnitine)	acylcarnitine
16	**C6:1**	Hexenoylcarnitine	acylcarnitine
17	**C7-DC**	Pimelylcarnitine	acylcarnitine
18	**C8**	Octanoylcarnitine	acylcarnitine
19	**C9**	Nonaylcarnitine	acylcarnitine
20	**C10**	Decanoylcarnitine	acylcarnitine
21	**C10:1**	Decenoylcarnitine	acylcarnitine
22	**C10:2**	Decadienylcarnitine	acylcarnitine
23	**C12**	Dodecanoylcarnitine	acylcarnitine
24	**C12-DC**	Dodecenoylcarnitine	acylcarnitine
25	**C12:1**	Dodecanedioylcarnitine	acylcarnitine
26	**C14**	Tetradecanoylcarnitine	acylcarnitine
27	**C14:1**	Tetradecenoylcarnitine	acylcarnitine
28	**C14:1-OH**	Hydroxytetradecenoylcarnitine	acylcarnitine
29	**C14:2**	Tetradecadienylcarnitine	acylcarnitine

30	**C14:2-OH**	Hydroxytetradecadienylcarnitine	acylcarnitine
31	**C16**	Hexadecanoylcarnitine	acylcarnitine
32	**C16-OH**	Hexadecenoylcarnitine	acylcarnitine
33	**C16:1**	Hydroxyhexadecenoylcarnitine	acylcarnitine
34	**C16:1-OH**	Hexadecadienylcarnitine	acylcarnitine
35	**C16:2**	Hydroxyhexadecadienylcarnitine	acylcarnitine
36	**C16:2-OH**	Hydroxyhexadecanoylcarnitine	acylcarnitine
37	**C18**	Octadecanoylcarnitine	acylcarnitine
38	**C18:1**	Octadecenoylcarnitine	acylcarnitine
39	**C18:1-OH**	Hydroxyoctadecenoylcarnitine	acylcarnitine
40	**C18:2**	Octadecadienylcarnitine	acylcarnitine
41	**Ala**	Alanine	amino acid
42	**Arg**	Arginine	amino acid
43	**Asn**	Asparagine	amino acid
44	**Asp**	Aspartate	amino acid
45	**Cit**	Citrulline	amino acid
46	**Gln**	Glutamine	amino acid

47	**Glu**	Glutamate	amino acid
48	**Gly**	Glycine	amino acid
49	**His**	Histidine	amino acid
50	**Ile**	Isoleucine	amino acid
51	**Leu**	Leucine	amino acid
52	**Lys**	Lysine	amino acid
53	**Met**	Methionine	amino acid
54	**Orn**	Ornithine	amino acid
55	**Phe**	Phenylalanine	amino acid
56	**Pro**	Proline	amino acid
57	**Ser**	Serine	amino acid
58	**Thr**	Threonine	amino acid
59	**Trp**	Tryptophan	amino acid
60	**Tyr**	Tyrosine	amino acid
61	**Val**	Valine	amino acid
62	**Ac-Orn**	Acetylornithine	biogenic amine
63	**ADMA**	Asymmetric dimethylarginine	biogenic amine

64	**alpha-AAA**	α-Aminoadipic acid	biogenic amine
65	**c4-OH-Pro**	cis-4-Hydroxyproline	biogenic amine
66	**Carnosine**	Carnosine	biogenic amine
67	**Creatinine**	Creatinine	biogenic amine
68	**DOPA**	Dihydroxyphenylalanine	biogenic amine
69	**Dopamine**	Dopamine	biogenic amine
70	**Histamine**	Histamine	biogenic amine
71	**Kynurenine**	Kynurenine	biogenic amine
72	**Met-SO**	Methionine sulfoxide	biogenic amine
73	**Nitro-Tyr**	Nitrotyrosine	biogenic amine
74	**PEA**	Phenylethylamine	biogenic amine
75	**Putrescine**	Putrescine	biogenic amine
76	**SDMA**	Symmetric dimethylarginine	biogenic amine
77	**Serotonin**	Serotonin	biogenic amine
78	**Spermidine**	Spermidine	biogenic amine
79	**Spermine**	Spermine	biogenic amine
80	**t4-OH-Pro**	trans-4-Hydroxyproline	biogenic amine

81	**Taurine**	Taurine	biogenic amine
82	**total DMA**	total dimethylarginine	biogenic amine
83	**lysoPC a C14:0**	lysoPhosphatidylcholine acyl C14:0	lysophosphatidylcholine
84	**lysoPC a C16:0**	lysoPhosphatidylcholine acyl C16:0	lysophosphatidylcholine
85	**lysoPC a C16:1**	lysoPhosphatidylcholine acyl C16:1	lysophosphatidylcholine
86	**lysoPC a C17:0**	lysoPhosphatidylcholine acyl C17:0	lysophosphatidylcholine
87	**lysoPC a C18:0**	lysoPhosphatidylcholine acyl C18:0	lysophosphatidylcholine
88	**lysoPC a C18:1**	lysoPhosphatidylcholine acyl C18:1	lysophosphatidylcholine
89	**lysoPC a C18:2**	lysoPhosphatidylcholine acyl C18:2	lysophosphatidylcholine
90	**lysoPC a C20:3**	lysoPhosphatidylcholine acyl C20:3	lysophosphatidylcholine
91	**lysoPC a C20:4**	lysoPhosphatidylcholine acyl C20:4	lysophosphatidylcholine
92	**lysoPC a C24:0**	lysoPhosphatidylcholine acyl C24:0	lysophosphatidylcholine
93	**lysoPC a C26:0**	lysoPhosphatidylcholine acyl C26:0	lysophosphatidylcholine
94	**lysoPC a C26:1**	lysoPhosphatidylcholine acyl C26:1	lysophosphatidylcholine
95	**lysoPC a C28:0**	lysoPhosphatidylcholine acyl C28:0	lysophosphatidylcholine
96	**lysoPC a C28:1**	lysoPhosphatidylcholine acyl C28:1	lysophosphatidylcholine
97	**PC aa C24:0**	Phosphatidylcholine diacyl C24:0	phosphatidylcholine

98	**PC aa C26:0**	Phosphatidylcholine diacyl C26:0	phosphatidylcholine
99	**PC aa C28:1**	Phosphatidylcholine diacyl C28:1	phosphatidylcholine
100	**PC aa C30:0**	Phosphatidylcholine diacyl C30:0	phosphatidylcholine
101	**PC aa C30:2**	Phosphatidylcholine diacyl C30:2	phosphatidylcholine
102	**PC aa C32:0**	Phosphatidylcholine diacyl C32:0	phosphatidylcholine
103	**PC aa C32:1**	Phosphatidylcholine diacyl C32:1	phosphatidylcholine
104	**PC aa C32:2**	Phosphatidylcholine diacyl C32:2	phosphatidylcholine
105	**PC aa C32:3**	Phosphatidylcholine diacyl C32:3	phosphatidylcholine
106	**PC aa C34:1**	Phosphatidylcholine diacyl C34:1	phosphatidylcholine
107	**PC aa C34:2**	Phosphatidylcholine diacyl C34:2	phosphatidylcholine
108	**PC aa C34:3**	Phosphatidylcholine diacyl C34:3	phosphatidylcholine
109	**PC aa C34:4**	Phosphatidylcholine diacyl C34:4	phosphatidylcholine
110	**PC aa C36:0**	Phosphatidylcholine diacyl C36:0	phosphatidylcholine
111	**PC aa C36:1**	Phosphatidylcholine diacyl C36:1	phosphatidylcholine
112	**PC aa C36:2**	Phosphatidylcholine diacyl C36:2	phosphatidylcholine
113	**PC aa C36:3**	Phosphatidylcholine diacyl C36:3	phosphatidylcholine
114	**PC aa C36:4**	Phosphatidylcholine diacyl C36:4	phosphatidylcholine

115	**PC aa C36:5**	Phosphatidylcholine diacyl C36:5	phosphatidylcholine
116	**PC aa C36:6**	Phosphatidylcholine diacyl C36:6	phosphatidylcholine
117	**PC aa C38:0**	Phosphatidylcholine diacyl C38:0	phosphatidylcholine
118	**PC aa C38:1**	Phosphatidylcholine diacyl C38:1	phosphatidylcholine
119	**PC aa C38:3**	Phosphatidylcholine diacyl C38:3	phosphatidylcholine
120	**PC aa C38:4**	Phosphatidylcholine diacyl C38:4	phosphatidylcholine
121	**PC aa C38:5**	Phosphatidylcholine diacyl C38:5	phosphatidylcholine
122	**PC aa C38:6**	Phosphatidylcholine diacyl C38:6	phosphatidylcholine
123	**PC aa C40:1**	Phosphatidylcholine diacyl C40:1	phosphatidylcholine
124	**PC aa C40:2**	Phosphatidylcholine diacyl C40:2	phosphatidylcholine
125	**PC aa C40:3**	Phosphatidylcholine diacyl C40:3	phosphatidylcholine
126	**PC aa C40:4**	Phosphatidylcholine diacyl C40:4	phosphatidylcholine
127	**PC aa C40:5**	Phosphatidylcholine diacyl C40:5	phosphatidylcholine
128	**PC aa C40:6**	Phosphatidylcholine diacyl C40:6	phosphatidylcholine
129	**PC aa C42:0**	Phosphatidylcholine diacyl C42:0	phosphatidylcholine
130	**PC aa C42:1**	Phosphatidylcholine diacyl C42:1	phosphatidylcholine
131	**PC aa C42:2**	Phosphatidylcholine diacyl C42:2	phosphatidylcholine

132	**PC aa C42:4**	Phosphatidylcholine diacyl C42:4	phosphatidylcholine
133	**PC aa C42:5**	Phosphatidylcholine diacyl C42:5	phosphatidylcholine
134	**PC aa C42:6**	Phosphatidylcholine diacyl C42:6	phosphatidylcholine
135	**PC ae C30:0**	Phosphatidylcholine acyl-alkyl C30:0	phosphatidylcholine
136	**PC ae C30:1**	Phosphatidylcholine acyl-alkyl C30:1	phosphatidylcholine
137	**PC ae C30:2**	Phosphatidylcholine acyl-alkyl C30:2	phosphatidylcholine
138	**PC ae C32:1**	Phosphatidylcholine acyl-alkyl C32:1	phosphatidylcholine
139	**PC ae C32:2**	Phosphatidylcholine acyl-alkyl C32:2	phosphatidylcholine
140	**PC ae C34:0**	Phosphatidylcholine acyl-alkyl C34:0	phosphatidylcholine
141	**PC ae C34:1**	Phosphatidylcholine acyl-alkyl C34:1	phosphatidylcholine
142	**PC ae C34:2**	Phosphatidylcholine acyl-alkyl C34:2	phosphatidylcholine
143	**PC ae C34:3**	Phosphatidylcholine acyl-alkyl C34:3	phosphatidylcholine
144	**PC ae C36:0**	Phosphatidylcholine acyl-alkyl C36:0	phosphatidylcholine
145	**PC ae C36:1**	Phosphatidylcholine acyl-alkyl C36:1	phosphatidylcholine
146	**PC ae C36:2**	Phosphatidylcholine acyl-alkyl C36:2	phosphatidylcholine
147	**PC ae C36:3**	Phosphatidylcholine acyl-alkyl C36:3	phosphatidylcholine
148	**PC ae C36:4**	Phosphatidylcholine acyl-alkyl C36:4	phosphatidylcholine

149	**PC ae C36:5**	Phosphatidylcholine acyl-alkyl C36:5	phosphatidylcholine
150	**PC ae C38:0**	Phosphatidylcholine acyl-alkyl C38:0	phosphatidylcholine
151	**PC ae C38:1**	Phosphatidylcholine acyl-alkyl C38:1	phosphatidylcholine
152	**PC ae C38:2**	Phosphatidylcholine acyl-alkyl C38:2	phosphatidylcholine
153	**PC ae C38:3**	Phosphatidylcholine acyl-alkyl C38:3	phosphatidylcholine
154	**PC ae C38:4**	Phosphatidylcholine acyl-alkyl C38:4	phosphatidylcholine
155	**PC ae C38:5**	Phosphatidylcholine acyl-alkyl C38:5	phosphatidylcholine
156	**PC ae C38:6**	Phosphatidylcholine acyl-alkyl C38:6	phosphatidylcholine
157	**PC ae C40:1**	Phosphatidylcholine acyl-alkyl C40:1	Phosphatidylcholine
158	**PC ae C40:2**	Phosphatidylcholine acyl-alkyl C40:2	phosphatidylcholine
159	**PC ae C40:3**	Phosphatidylcholine acyl-alkyl C40:3	phosphatidylcholine
160	**PC ae C40:4**	Phosphatidylcholine acyl-alkyl C40:4	phosphatidylcholine
161	**PC ae C40:5**	Phosphatidylcholine acyl-alkyl C40:5	phosphatidylcholine
162	**PC ae C40:6**	Phosphatidylcholine acyl-alkyl C40:6	phosphatidylcholine
163	**PC ae C42:0**	Phosphatidylcholine acyl-alkyl C42:0	phosphatidylcholine
164	**PC \ae C42:1**	Phosphatidylcholine acyl-alkyl C42:1	phosphatidylcholine
165	**PC ae C42:2**	Phosphatidylcholine acyl-alkyl C42:2	phosphatidylcholine

166	**PC ae C42:3**	Phosphatidylcholine acyl-alkyl C42:3	phosphatidylcholine
167	**PC ae C42:4**	Phosphatidylcholine acyl-alkyl C42:4	phosphatidylcholine
168	**PC ae C42:5**	Phosphatidylcholine acyl-alkyl C42:5	phosphatidylcholine
169	**PC ae C44:3**	Phosphatidylcholine acyl-alkyl C44:3	phosphatidylcholine
170	**PC ae C44:4**	Phosphatidylcholine acyl-alkyl C44:4	phosphatidylcholine
171	**PC ae C44:5**	Phosphatidylcholine acyl-alkyl C44:5	phosphatidylcholine
172	**PC ae C44:6**	Phosphatidylcholine acyl-alkyl C44:6	phosphatidylcholine
173	**SM (OH) C14:1**	Hydroxysphingomyeline C14:1	sphingolipid
174	**SM (OH) C16:1**	Hydroxysphingomyeline C16:1	sphingolipid
175	**SM (OH) C22:1**	Hydroxysphingomyeline C22:1	sphingolipid
176	**SM (OH) C22:2**	Hydroxysphingomyeline C22:2	sphingolipid
177	**SM (OH) C24:1**	Hydroxysphingomyeline C24:1	sphingolipid
178	**SM C16:0**	Sphingomyeline C16:0	sphingolipid

179	**SM C16:1**	Sphingomyeline C16:1	sphingolipid
180	**SM C18:0**	Sphingomyeline C18:0	sphingolipid
181	**SM C18:1**	Sphingomyeline C18:1	sphingolipid
182	**SM C20:2**	Sphingomyeline C20:2	sphingolipid
183	**SM C22:3**	Sphingomyeline C22:3	sphingolipid
184	**SM C24:0**	Sphingomyeline C24:0	sphingolipid
185	**SM C24:1**	Sphingomyeline C24:1	sphingolipid
186	**SM C26:0**	Sphingomyeline C26:0	sphingolipid
187	**SM C26:1**	Sphingomyeline C26:1	sphingolipid
188	**H1**	Sum of Hexoses (90% glucose)	sugar

Supplementary Table S2: Demographics of archival samples

Sample group	Mean age/years	Primigravida/%	Multigravida/%	1st trimester	2nd trimester	3rd trimester
Malaria only	25.5	32.5	67.5	30	57.5	12.5
CHB only	26.7	23.5	76.5	45.6	47.1	7.4
Malaria+CHB	26.9	25	75	33.3	58.3	8.3
Uninfected	27.6	16.4	83.3	35.6	46.6	17.8
P-value (95% confidence interval)	0.099		0.1466			0.257

Supplementary Table S3: Table Venn diagram results showing the metabolite elements shared among the disease groups

Comparison groups	Total metabolites altered	Metabolite elements
Co-infected & Hep B & Malaria	13	PC ae C44:4 SM (OH) C22:1 SM C16:0 SM C22:3 SM (OH) C16:1 SM (OH) C14:1 PC aa C28:1 PC aa C40:5 SM C16:1 SM C24:0 SM (OH) C22:2 PC aa C38:5 SM C24:1
Hep B & Malaria	1	PC aa C32:0
Co-infected & Malaria	4	PC aa C38:6 SM C18:0 SM (OH) C24:1 SM C18:1
Co-infected & Hep B	52	PC ae C42:2 PC ae C40:1 PC ae C42:3 PC aa C32:3 PC aa C34:4 PC ae C42:5 PC ae C36:2 PC ae C36:5 Trp PC aa C36:4 PC ae C30:2 PC aa C40:1 PC aa C42:4 PC ae C42:4 PC aa C40:3 PC aa C26:0 PC aa C40:2 PC aa C38:1 PC aa C24:0 PC aa C34:3 PC aa C36:3 PC aa C32:2 PC ae C44:6 PC ae C36:3 PC aa C34:1 lysoPC a C26:1 PC ae C38:4 PC ae C34:3 PC ae C40:6 PC aa C38:3 PC ae C40:3 H1 PC ae C38:3 PC ae C34:2 PC ae C44:5 PC aa C40:4 PC ae C38:2 PC ae C40:4 PC aa C36:2 PC aa C42:5 PC ae C40:2 PC aa C42:6 PC ae C42:1 PC aa C42:1 PC ae C38:1 PC aa C42:2 PC ae C36:1 PC aa C36:1 PC ae C42:0 PC ae C40:5 PC aa C34:2 PC aa C38:4
Malaria	4	C14:1 Lys Thr C16:1-OH
Hep B	18	Asp Phe PC aa C42:0 PC ae C38:5 lysoPC a C24:0 Kynurenine lysoPC a C18:0 lysoPC a C28:0 PC aa C30:0 PC ae C36:4 C4 PC ae C34:0 Pro C4:1 Serotonin Arg C6:1 PC ae C30:1
Co-infected	13	C5-OH (C3-DC-M) PC aa C40:6 PC aa C36:6 SM C20:2 PC aa C36:5 PC aa C36:0 PC ae C44:3 C5-M-DC PC aa C38:0 PC ae C38:0 Val PC ae C38:6 PC ae C32:2

Supplementary Table S4: Area Under the Curve (AUC) values for univariate biomarker analysis in the malaria vs uninfected groups

Biomarker	AUC	P-value from T-test	Log 2 Fold Change	Cluster
PC aa C28:1/PC aa C32:0	0.756031	9.52E-07	0.16535	2
PC aa C32:0/SM (OH) C22:2	0.742599	0.000355	0.95456	5
C16:1-OH/SM (OH) C22:2	0.739583	0.000612	-0.09203	3
PC aa C32:0/PC ae C44:4	0.735197	4.32E-05	0.11374	5
PC aa C32:0/SM (OH) C16:1	0.724507	0.000618	0.24042	5
PC aa C32:0/PC aa C38:5	0.723684	2.41E-05	-0.61225	5
PC aa C32:0/SM (OH) C14:1	0.723684	0.000617	0.32978	5
Lys/PC aa C32:0	0.71875	1.17E-05	-0.12705	2
C16:1-OH/PC aa C38:5	0.715186	0.000121	-0.06976	3
C16:1-OH/SM C22:3	0.715186	4.53E-05	-0.35812	3
C16:1-OH/PC aa C28:1	0.709978	5.20E-05	-0.11246	3
C16:1-OH/Lys	0.706826	4.92E-05	-0.04493	3
C16:1-OH/SM (OH) C16:1	0.705866	0.000645	-0.1053	3
C16:1-OH/PC aa C40:5	0.703673	0.000454	-0.07683	3
PC aa C32:0/PC aa C40:5	0.70148	6.75E-05	0.92344	5

AUC cutoff >0.7

Supplementary Table S5: Area Under the Curve (AUC) values for univariate biomarker analysis in the Hepatitis B vs uninfected groups

Biomarker	AUC	P-value from T-test	Log 2 Fold Change	Cluster
Asp	0.748736	5.29E-06	-0.67548	4
Phe	0.73966	0.008831	-0.20458	4
PC aa C36:2	0.727252	3.33E-05	-0.32898	2
SM C24:0	0.723346	4.57E-06	-0.31286	2
PC aa C40:1	0.715188	0.000282	-0.35356	5
PC aa C40:4	0.709674	2.37E-05	-0.38036	2
PC ae C44:4	0.70795	3.24E-05	-0.38927	2
PC aa C38:4	0.705193	2.92E-05	-0.36599	3
PC ae C42:4	0.704159	3.31E-05	-0.3797	2

AUC cutoff >0.7

Supplementary Table S6: Area Under the Curve (AUC) values for univariate biomarker analysis in the Co-infected vs uninfected groups

Biomarker	AUC	P-value from T-test	Log 2 Fold Change	Cluster
PC ae C40:1	0.801649	5.91E-05	-99	2
PC ae C42:3	0.792318	1.65E-05	1.7544	2
PC aa C42:5	0.785807	0.000568	0.47364	2
SM C24:1	0.777778	1.97E-06	-0.09774	5
SM (OH) C22:2	0.776259	2.16E-06	-0.32341	5
PC ae C42:5	0.775608	0.000245	-0.5096	2
PC aa C40:1	0.770833	0.003732	0.47242	2
PC aa C28:1	0.767578	3.34E-05	1.6285	1
SM C24:0	0.766927	6.06E-06	-0.12553	5
PC aa C32:3	0.759766	0.002364	0.26904	2
PC ae C42:4	0.756944	0.003386	-1.8317	2
PC ae C42:2	0.754123	0.004381	0.48377	2
PC ae C42:3/PC ae C34:2	0.748698	2.84E-05	0.18265	3
PC aa C42:6	0.74783	0.000665	0.58209	4
SM (OH) C24:1	0.746962	0.000192	0.92823	5
PC aa C40:4	0.746962	0.000406	-0.36234	4
PC aa C42:1	0.746745	0.000908	0.28211	4
SM (OH) C22:1	0.744575	0.000942	-0.17261	5
SM (OH) C22:1.1	0.744575	0.000942	-0.17261	5
PC aa C40:3	0.743273	0.001578	1.3363	2
PC ae C40:6	0.742405	0.001496	-0.39989	4
SM C16:0	0.742405	0.001702	-0.06236	5
PC aa C42:4	0.738498	0.018837	0.59855	2
PC ae C40:4	0.73763	0.006181	-0.36582	2
PC ae C40:1/PC aa C36:1	0.737413	0.001521	0.096368	3
PC ae C40:5	0.736545	0.002143	-0.30212	2
PC aa C24:0	0.735677	0.000111	0.41141	1
PC ae C42:0	0.735026	0.006807	0.48461	4
PC ae C42:3/PC aa C36:1	0.734809	0.000214	0.10182	3
PC ae C40:1/PC ae C34:2	0.733507	0.0003	0.18636	3
SM C20:2	0.728733	0.001923	0.25816	5
PC ae C42:3/PC ae C36:3	0.727865	0.000161	0.23139	3
PC ae C40:2	0.726563	0.026784	-1.9027	2
SM (OH) C14:1	0.723741	0.000128	-0.39355	5
PC ae C40:1/PC aa C36:2	0.721354	0.000488	0.074368	3
PC ae C40:1/PC ae C36:3	0.72092	0.001116	0.24842	3
SM C18:0	0.720486	0.000168	-0.14951	5
PC aa C38:1	0.720269	0.006709	1.1939	2

Biomarker	AUC	P-value from T-test	Log 2 Fold Change	Cluster
PC ae C42:3/PC aa C36:2	0.720052	0.000137	0.079704	3
PC aa C36:6	0.71875	0.001209	0.86211	2
SM (OH) C16:1	0.717231	9.61E-05	-0.94976	5
PC ae C42:3/PC aa C38:0	0.716146	0.000274	0.3782	3
PC aa C36:0	0.71441	0.165218	-0.5834	2
SM C16:1	0.71224	0.010459	-0.13557	5
PC aa C38:5	0.712023	0.001268	-0.12753	4
PC ae C44:4	0.711806	0.01122	0.24332	4
PC ae C42:3/PC ae C32:2	0.711372	0.000146	-0.47905	3
PC aa C38:3	0.709201	0.016079	-0.0896	4
PC ae C42:3/PC aa C34:1	0.709201	0.000127	0.06871	3
PC aa C34:3	0.70855	0.001508	-0.30132	2
PC ae C38:4	0.705512	0.025892	-0.15552	4
PC ae C36:1	0.702691	0.003868	-0.23862	2
PC ae C42:3/PC ae C38:6	0.702691	0.00054	0.23177	3
PC ae C42:1	0.702257	0.004484	0.44241	2
PC aa C36:3	0.702257	0.008219	-0.06669	4
PC ae C38:0	0.700087	0.006332	-0.64977	2

AUC cutoff >0.7
